# SEM, TEM, and IHC Analysis of the Sinus Node and Its Implications for the Cardiac Conduction System

**DOI:** 10.1155/2013/961459

**Published:** 2013-10-27

**Authors:** D. Mandrioli, F. Ceci, T. Balbi, C. Ghimenton, G. Pierini

**Affiliations:** ^1^Ramazzini Institute, Cesare Maltoni Cancer Research, 40010 Bentivoglio (Bologna), Italy; ^2^Service of Nuclear Medicine, Department of Radiological and Histopathological Sciences, S. Orsola Malpighi Hospital, University of Bologna, 40138 Bologna, Italy; ^3^Department of Surgical Pathology, S. Orsola Malpighi Hospital, 40138 Bologna, Italy; ^4^Department of Surgical Pathology, Borgo Trento Hospital, 37126 Verona, Italy; ^5^Department of Forensic Medicine, University of Bologna, 40126 Bologna, Italy

## Abstract

More than 100 years after the discovery of the sinus node (SN) by Keith and Flack, the function and structure of the SN have not been completely established yet. The anatomic architecture of the SN has often been described as devoid of an organized structure; the origin of the sinus impulse is still a matter of debate, and a definite description of the long postulated internodal specialized tract conducting the impulse from the SN to the atrioventricular node (AVN) is still missing. In our previously published study, we proposed a morphologically ordered structure for the SN. As a confirmation of what was presented then, we have added the results of additional observations regarding the structural particularities of the SN. We investigated the morphology of the sinus node in the human hearts of healthy individuals using histochemical, immunohistochemical, optical, and electron microscopy (SEM, TEM). Our results confirmed that the SN presents a previously unseen highly organized architecture.

## 1. Introduction

The first observation of the SN structure dates back to 1910, when Arthur Keith and Martin Flack introduced the world to the location of the sinus node (SN), observing that “we noted this structure, but attached no functional meaning to it” [1, 2]. A century after its discovery, the structure and function of the SN still remain a mystery which has yet to be unfolded. To the best of our knowledge, our previous published study on the architecture of the SN was the first to propose a model with a morphologically ordered structure [3].

First of all, the anatomic architecture of the SN has often been described as devoid of a definitive shape or an organized structure [4–10]. The results of the 3D reconstructions of the atrial elements [11–14] and the mathematical [15, 16] and ultrastructural models [17] of the SN are strongly divergent. The fact that the SN presents a different shape in humans, as compared to other mammals, complicates the task of creating a reliable model of this structure even more [6].

Second, the origin of the sinus impulse is still a matter of debate: what triggers it? Research in the fields of electrophysiology [18] and molecular biology [19–24] has pointed out that ion channels and intracellular Ca^2^ signalling are necessary for the proper setting of a pacemaker mechanism [25–38]. Brain-type Na channels have also recently been discovered, but their role is still unclear [39–41]. Sinus node automaticity is not fully understood, but it seems to arise from a dynamic balance between positive inward currents which favour depolarization and positive outward currents [42] which promote repolarization [43]. Depolarizing currents are indeed involved in highly stable phase-4 depolarization and pacemaking [15]. Each of these currents provides a potential target for pacemaker regulation [44]. Nevertheless, these currents are not sufficient to explain the pacemaking function of the SN; in particular, they fail to explain the synchronous depolarization mechanism of the sinoatrial cells [45, 46]. Mangoni and Nargeot have clearly synthesized this complex question in their recent complete review confirming that it is still not completely clear, for example, which ion channels are essential for generating diastolic depolarization in the SN, atrioventricular node (AVN), and the Purkinje fibers network and which mechanisms play a dominant role in the autonomic regulation of automaticity in humans [25].

Third, anatomists and pathologists are still not in agreement regarding the internodal tract conducting the impulse from the SN to the AVN. According to Anderson and Ho, “There is no evidence of morphologically specialized tracts between the sinus and the atrioventricular nodes” [4] and they proposed “preferential conduction more likely reflects the arrangement of the working internodal cells and their related cellular properties.” There has been much research carried out in vain in order to identify this “preferential conduction” cellular pathway. Cells expressing HCN4 and Cx45 [23, 46] in the atrium have recently been proposed as candidates for SN to AVN preferential conduction, but the authors also acknowledge that “There was not a continuous tract of HCN4-expressing cells between the SN and the AVN” [46].

For those reasons, the aim of the current study was to investigate the morphology of the SN in depth, integrating standard histological procedures with SEM and TEM microscopy, histochemistry, and immunohistochemistry. Moreover, together with the standard SN section technique based on orthogonal cutting on the SN artery plane, we introduced a new slicing approach, sectioning the SN parallel to the SN artery. 

## 2. Materials and Methods

The collection technique of the SN and the procedures for the examination of the cardiac conduction system using optic and electronic microscopy are identical to those described in the previous communication [1]. 

Twenty-five autoptic cases in which neither pathologic modifications of the conduction system nor dysplastic modifications of the SN artery were present were chosen. The SN was examined according to the procedure suggested by Balbi et al. [3]; the specimens were sectioned perpendicular to the terminal crest and fixed in FineFix (Milestone, Bergamo, Italy) and microwave-processed (ATP1, Kaltek, Italy). The tissues were paraffin-embedded.

For histology, staining with hematoxylin-eosin and luxol fast blue was carried out; observations were conducted with direct and polarized light; for histochemistry, toluidine blue and orcein were used (all reagents from Histo-Line laboratories, Milano, Italy). For immunohistochemistry, c-kit (CD117), vimentin, S100, *α*-tubulin, synaptophysin, neurofilaments, calretinin, desmin, calcitonin, and Ck AE1-AE3 were tested as were the following neuroendocrine markers: serotonin, somatostatin, chromogranin, and neuron specific enolase (all reagents are from NBL Int).

For scanning electron microscopy (SEM), fixed material was dehydrated in an alcohol-ascending series for preliminary drying, then critical-point-dried, and finally gold-coated (50 A thickness). A SEM Philips 505 equipped with a backscattered detector and digital image recording was used.

For transmission electron microscopy (TEM), sinoatrial tissue was carefully recovered from paraffin blocks; the samples were dewaxed in xylene and rehydrated; after fixation with 1% buffered osmium tetroxide (Histo-Line laboratories, Milano, Italy), the samples were dehydrated in alcohol and embedded in epoxy resin; 60 to 80 nm thick sections were stained with uranyl acetate and lead citrate and were examined under a Philips 400T transmission electron microscope (reagents are from C.Erba, Milano, Italy).

## 3. Results

**Figure 1:**
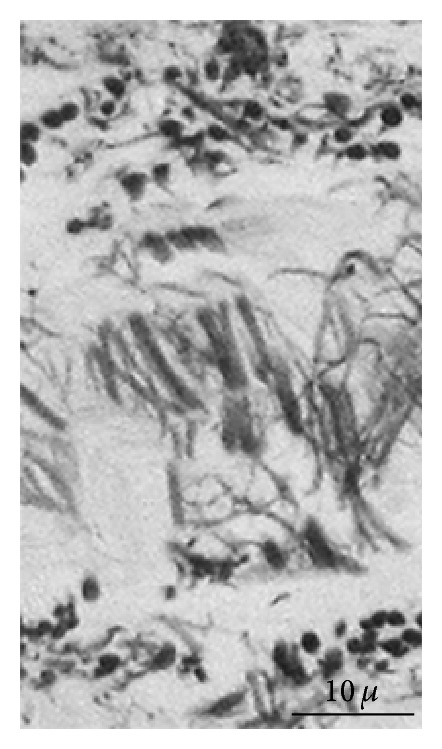
SN architecture. Elastic fibers geometrically arranged to form contiguous compartments; these fibers suggest a structure with orthogonally oriented walls (histochemistry: orcein HCl, ×800).

**Figure 2:**
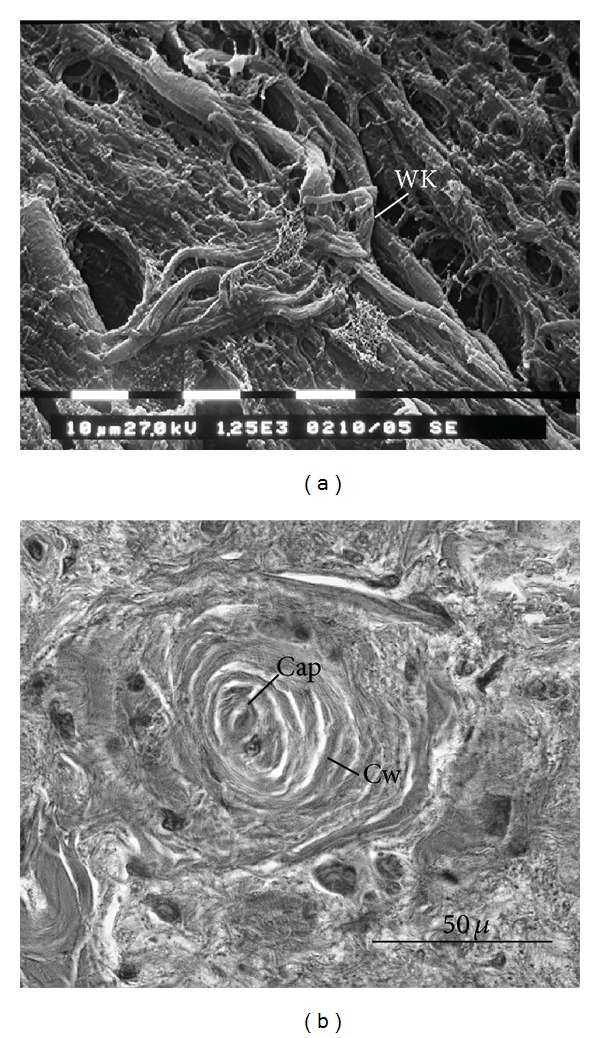
SN architecture. (a) Sections longitudinal to the SN artery; it is easy to appreciate the inside structure of the cage and the knots at its vertices (SEM ×1250) (Sl: fibroblast-like cell, WK: elastic cage knot). (b) Sections throughout the artery plane showing cages as vortices (Cap: capillary vessel, Cw: cage wall; HE ×600).

**Figure 3:**
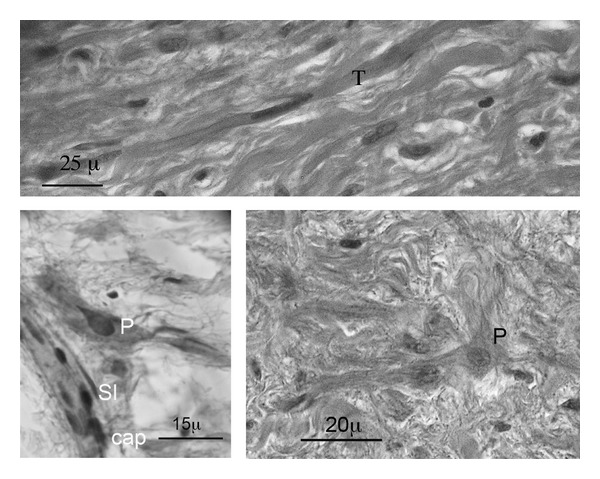
Sinus node (SN) cells. The SN typical cell types (P: pale cells, T: transitional cells, Sl: fibroblast-like cells, and Cap: capillary vessel; HE ×600).

**Figure 4:**
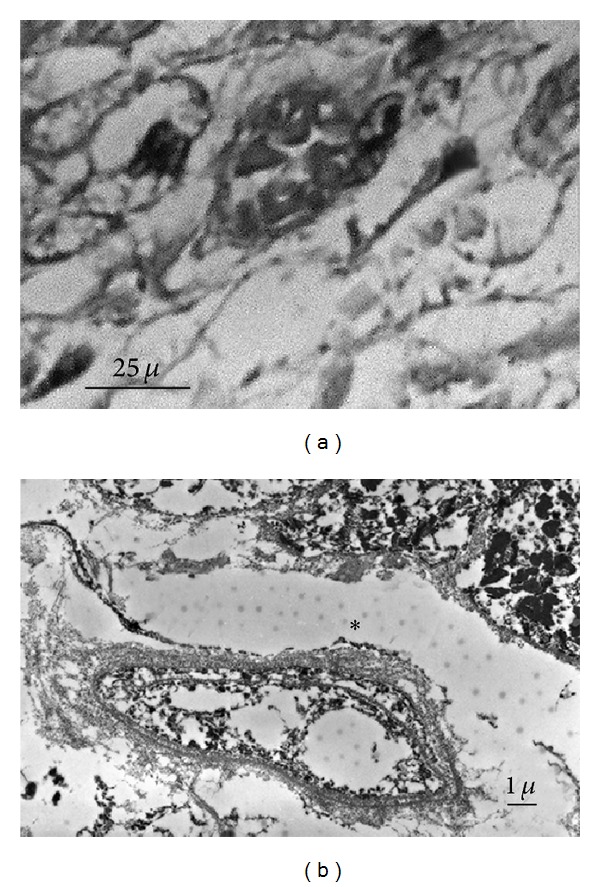
Fibroblast-like cells. (a) Thin cell processes interconnected with each other; the cellular extensions are revealed by dark dots (HE ×600). (b) Dots on expanded fibroblast-like extensions (TEM ×10.000).

**Figure 5:**
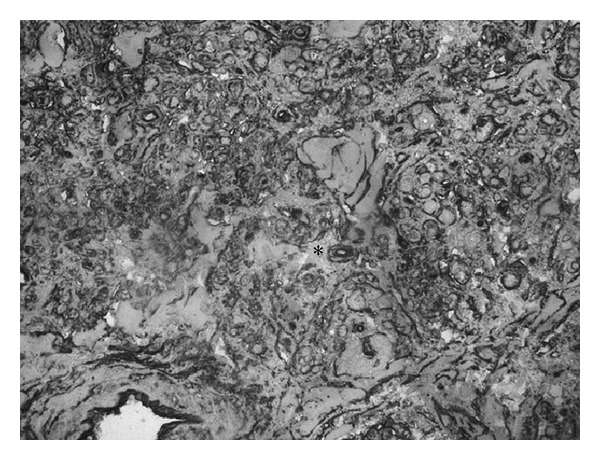
CD34 expression in the cell population of the SN (ICH, CD 34, *red* color, ×400).

**Figure 6:**
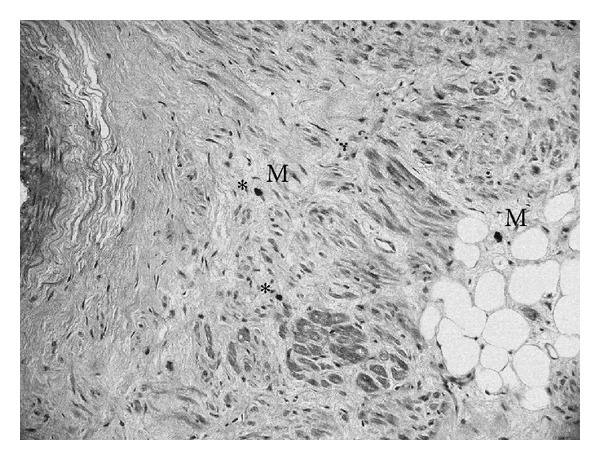
Mast cells of the SN (toluidine blue, ×400).

Our work based on new anatomical samples confirmed the evidence of the presence of regular periodic architecture in the SN. Elastic and connective fibres that surround the inner SN artery as quadrangular chambers are approximately 120 microns long. The major axis of these chambers is arranged parallel to the inner SN artery (Figure 1). The most recent data now demonstrate that, at the vertices of these polyhedrons, the connective and elastic fibres are interlaced, forming a “hook-like” closure (Figure 2). Three types of cells were confirmed to be present within the chambers: P (pale) cells, T (transitional) cells, and fibroblast-like cells. Pale cells are spherical and/or star-shaped with long cytoplasmic processes; only P cells are randomly disposed within the matrix of the cages (Figure 3). The T cells are similar to myocytes but with a reduced number of sarcomeres; T cells cover the internal perimeter of the cages, both horizontally and vertically. Fibroblast-like cells show long multipolar extensions making contact with other cells, vessels, and connective tissues forming a thin three-dimensional network (Figure 4). Immunohistochemistry showed that the three cell types of the SN expressed mesenchymal markers indicative of their embryological origin when stained with CD34 (Figure 5), a 110 KDa transmembrane glycoprotein of unknown function expressed by progenitor hemopoietic cells, interstitial Cajal cells, and myofibroblastic soft tissues. Random activated mast-cells are also present (Figure 6).

**Figure 7:**
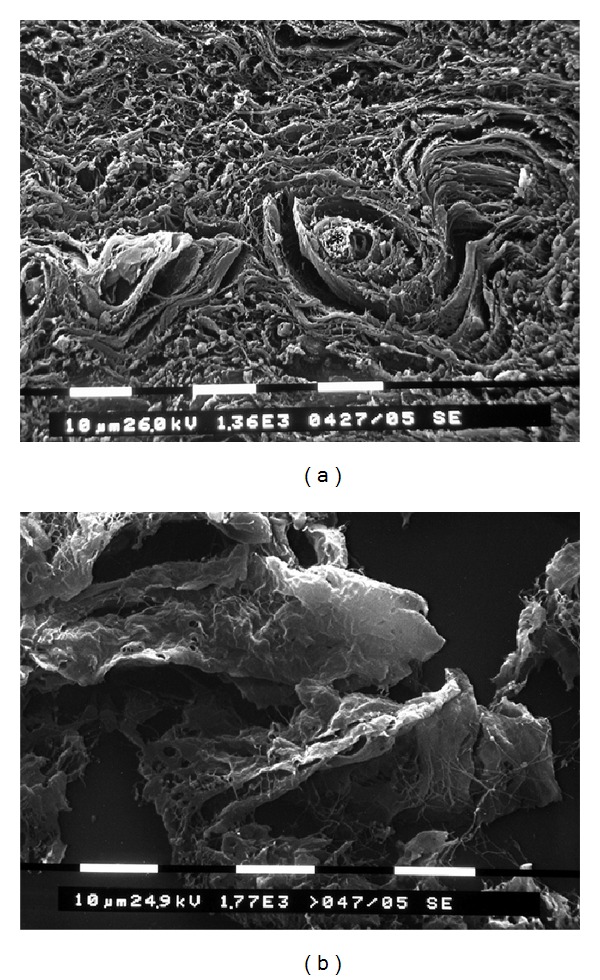
SN inner structure. In the center, helicoidal connective structures can be seen in sections parallel to the SN artery, regularly twisted whereas, in the transverse sections, they appear as vortices (on the left, note the inner centered capillary (*) and on the right, note SEM ×925, ×1360, and ×1770).

**Figure 8:**
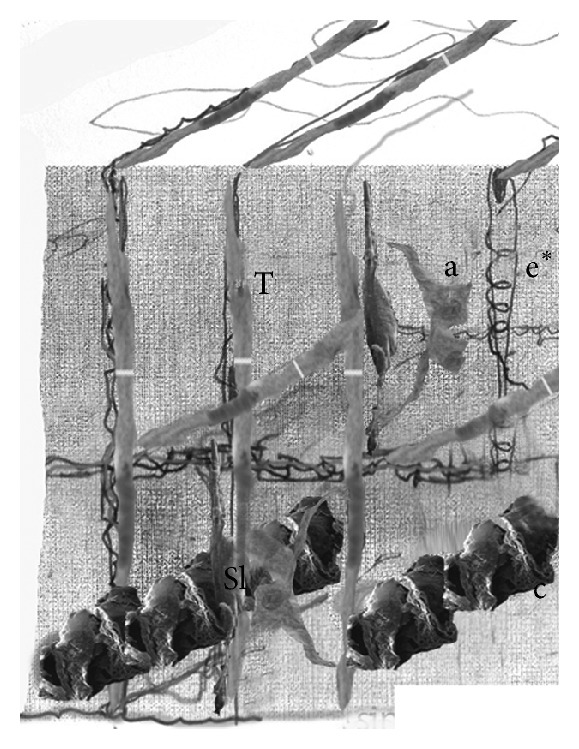
Artistic rendering of the whole SN structure.

A helicoid made up of thick and firm connective tissue, always located in the middle of the cage, contains a thin capillary vessel at its centre having its own elastic ring (Figure 7).  Figure 8 shows an artistic rendering of the SN chamber reconstruction according to our observations.

## 4. Discussion

Our results confirm that the SN has a well-organized anatomic structure. It presented unique and differentiated characteristics as compared to the rest of the atrial myocardia. The presence of numerous predominantly activated mast cells, together with their own neural innervation, could make the SN an appropriate and suitable structure to integrate the humoral and neural stimuli which regulate the heartbeat. The strong presence of mast cells in the SN is likely responsible for some of the clinical effects of histaminic dysregulation such as the presence of antihistaminic drugs and anaphylactic shock which can induce alterations of the heartbeat [47–49]. Our observations could open a new perspective as how to answer Mangoni's question concerning “which mechanism plays a dominant role in the autonomic regulation of automaticity in humans.” An ordinate and coordinate structure of the SN, as we described, could perhaps be an introduction to novel research in order to better understand this automaticity rather than the current model based on single independent cells with their own automaticity. In fact, according to this proposed model, the SN cells are supposed to undergo spontaneous depolarization; the fastest are the primary pacemaker cells of the heart which determine the heart rate [25].

Until now, this model has been considered necessary and sufficient. Anyway, even if the necessary depolarization of the SN cells in order to create the impulse is not under discussion and is strongly evidence based, the sufficiency criteria of this model, namely, that single cells can impose their rhythm due to their faster rate, seem to be challenged by both clinical evidence and our results. Is the structure itself, rather than the single cells, sufficient to generate a harmonic and coordinated heartbeat? Could it play a role in SN function, perhaps coupling the contiguous chambers electromagnetically? Could it be important in the forensic study of sudden death? 

In fact, an electrical depolarization of the entire atrium constantly follows SN electrical depolarization. Otherwise, it may be worth considering that a magnetic pulse is also constantly generated with each electrical pulse; the heartbeat generates a magnetic field, which is the strongest magnetic field of the human body (100 pT) [50, 51]. Also external electromagnetic fields have been shown to markedly affect heart function in experimental animals [52]. 

The current work was designed to demonstrate and confirm the order in the structure of the SN. This confirmation could induce us to hypothesize an important role of this coupled and organized structure, and not exclusively of the single cells of the SN, in the generation and conduction of the electromagnetic cardiac pulse.
